# Irisin and troponin I expression in dialysis patients submitted to remote ischemic preconditioning: a pilot study

**DOI:** 10.1590/2175-8239-JBN-2019-0051

**Published:** 2019-12-02

**Authors:** Flávia de Sousa Gehrke, Mariana Carvalho Gouveia, Carla Gabriela Marques Barbosa, Neif Murad, Beatriz da Costa Aguiar Alves Reis, Fernando Luiz Affonso Fonseca, Edimar Cristiano Pereira, Marcelo Rodrigues Bacci

**Affiliations:** 1 Universidade Paulista, Departamento de Farmácia, São Paulo, SP, Brasil.; 2 Instituto de Assistência Médica ao Servidor Público Estadual, Programa de Pós-Graduação em Ciências da Saúde, São Paulo, SP, Brasil.; 3 Centro Universitário em Saúde do ABC, Departamento de Nefrologia, Santo André, SP, Brasil.; 4 Centro Universitário em Saúde do ABC, Departamento de Cardiologia, Santo André, SP, Brasil.; 5 Centro Universitário em Saúde do ABC, Laboratório de Análises Clínicas, Santo André, SP, Brasil.; 6 Universidade Federal de São Paulo, Departamento de Ciências Farmacêuticas, Diadema, SP, Brasil.

**Keywords:** Renal Dialysis, Hypertension, Diabetes Mellitus, Blood Chemical Analysis, Troponin I, Diálise Renal, Hipertensão, Diabetes Mellitus, Análise Química do Sangue, Troponina I

## Abstract

**Background:**

Renal replacement therapy continues to be related to high hospitalization rates and poor quality of life. All-cause morbidity and mortality in renal replacement therapy in greater than 20% per year, being 44 times greater when diabetes is present, and over 10 times that of the general population. Regardless of treatment, the 5-year survival is 40%, surpassing many types of cancers. Irisin is a hormone that converts white adipose tissue into beige adipose tissue, aggregating positive effects like fat mass control, glucose tolerance, insulin resistance, prevention of muscle loss, and reduction in systemic inflammation.

**Objectives:**

To determine the serum levels of troponin I in hemodialysis patients submitted to remote ischemic preconditioning (RIPC) associated with irisin expression.

**Methods:**

This was a prospective, randomized, double-blind clinical trial with patients with chronic kidney disease submitted to hemodialysis for a 6-month period. Troponin I, IL-6, urea, TNF-α, and creatinine levels were determined from blood samples. The expressions of irisin, thioredoxin, Nf-kb, GPX4, selenoprotein and GADPH were also evaluated by RT-PCR.

**Results:**

Samples from 14 hypertensive patients were analyzed, 9 (64.3%) of whom were type 2 diabetics, aged 44-64 years, and 50% of each sex. The difference between pre- and post-intervention levels of troponin I was not significant. No differences were verified between the RIPC and control groups, except for IL-6, although a significant correlation was observed between irisin and troponin I.

**Conclusion:**

Remote ischemic preconditioning did not modify irisin or troponin I expression, independent of the time of collection.

## RESUMO

**Introdução:**

A terapia de substituição renal continua associada a altas taxas de hospitalização e baixa qualidade de vida. A morbimortalidade por todas as causas na terapia de substituição renal é superior a 20% ao ano, sendo 44 vezes maior quando a diabetes está presente e mais de 10 vezes a da população em geral. Independentemente do tratamento, a sobrevida em 5 anos é de 40%, superando muitos tipos de câncer. A irisina é um hormônio que converte tecido adiposo branco em tecido adiposo bege, agregando efeitos positivos como o controle de massa gorda, tolerância à glicose, resistência à insulina, prevenção de perda muscular e redução da inflamação sistêmica.

**Objetivos:**

Determinar os níveis séricos de troponina I em pacientes em hemodiálise submetidos ao pré-condicionamento isquêmico remoto (PCIR) associado à expressão da irisina.

**Métodos:**

Estudo clínico prospectivo, randomizado, duplo-cego, com pacientes com doença renal crônica submetidos à hemodiálise por um período de 6 meses. Os níveis de troponina I, IL-6, uréia, TNF-α e creatinina foram determinados a partir de amostras de sangue. As expressões de irisina, tioredoxina, Nf-kb, GPX4, selenoproteína e GADPH foram também avaliadas por RT-PCR.

**Resultados:**

Foram analisadas amostras de 14 pacientes hipertensos, 9 (64,3%) dos quais eram diabéticos tipo 2, com idades entre 44 e 64 anos e 50% de cada gênero. A diferença entre os níveis pré e pós-intervenção de troponina I não foi significativa. Não houve diferenças entre os grupos PCIR e controle, exceto pela IL-6, embora tenha sido observada correlação significativa entre irisina e troponina I.

**Conclusão:**

O pré-condicionamento isquêmico remoto não modificou a expressão de irisina ou troponina I, independentemente do tempo de coleta.

## INTRODUCTION

Despite the recent improvements in the dialysis process, renal replacement therapy continues to present high hospitalization rates, which is related to poor quality of life. All-cause morbidity and mortality in renal replacement therapy is greater than 20% per year, being 44 times greater when diabetes is present and over 10 times that of the general population^1^^,^ 2. Regardless of treatment, the 5-year survival is 40%, which surpasses that of numerous types of cancers^3^^,^ 4. In hemodialysis patients, cardiovascular mortality accounts for 40% of all deaths, mostly due to heart failure, acute myocardial infarction, and fatal cardiac arrhythmia^3^^,^ 5. During prolonged treatment, these patients are susceptible to morphofunctional alterations. Quantification of myocardial blood flow (MBF) by positron emission tomography (PET) during hemodialysis of patients with no significant angiographic coronary lesion evidence alterations in the left ventricular (LV) segmental contraction that were correlated with the reduction in both global and segmental MBF, promoting contractile dysfunction^6^. Reduction in segmental MBF is associated with circumscribed areas of necrosis, altered permeability, elevated circulating levels of cardiac troponin (cTnT), together with LV hypokinetic regions detected by echocardiography. Recurrent ischemic episodes during hemodialysis promote myocardial injury with functional irreversibility^6^.

Myocardial ischemia can be triggered by several factors: high prevalence of coronary atheroma, left ventricular hypertrophy, intradialytic hypotension, and reduced reserve coronary flow (RRF), even in the absence of stenosis^7^^,^ 8. Left ventricular hypertrophy (LVH), which often occurs in renal failure, increases the ventricular end-diastolic pressure, the parietal stress that compromises MBF, particularly in the subendocardium^9^. Myocardial stunning of LV dysfunction due to transient ischemia associated with hemodialysis is frequently prolonged but reversible^6^^,^ 10. Ischemic episodes due to hemodialysis play an important role in the development of heart failure and cardiac arrhythmias^11^^,^ 12. Thus, reducing ischemia following hemodialysis seems to be a desirable therapeutic target^13^.

Irisin is a hormone identified in muscle cells of transgenic mice expressed by Ppargc1a, which encodes the co-activator-1α of the γ receptor activated by peroxisome proliferator (PGC-1α). In turn, PGC-1α stimulates the gene expression of the transmembrane protein fibronectin type III domain-containing protein 5 (Fndc5). When Fndc5 undergoes proteolytic cleavage, it is released into the bloodstream with a fragment containing 112 residual amino acids. It binds to unidentified receptors on the cell surface of adipose tissue^14^. Irisin converts white adipose tissue into beige adipose tissue, which aggregates positive effects like fat mass control, glucose tolerance, insulin resistance, prevention of muscle loss, and reduction in systemic inflammation^15^^,^ 16. Irisin has the therapeutic potential to prevent and treat obesity and diabetes^17^. In humans, FNDC5 is strongly expressed in skeletal muscle, heart, tongue, and rectum. FNDC5 expression is decreased in the pancreas, liver, and organs involved in glycolytic homeostasis^18^. In humans, FNDC5 expression in adipose tissue is up to 200 times lower than that of skeletal muscle^18^^,^ 19. Circulating levels are modulated by factors that include diet, obesity, exercise, pharmacological agents, and different pathological conditions. Remote ischemic preconditioning (RIPC) is a non-invasive, non-pharmacological intervention of myocardial protection induced by transient interruption of the blood flow in one limb by a blood pressure cuff, which shows a protective effect against myocardial ischemia (reperfusion injury)^18^^,^ 20 ^,^ 21. RIPC is associated with a reduction in troponin I release, lower elevation in the ST segment of the ECG, and lower adverse cardiovascular events following percutaneous coronary intervention (PCI)^19^^,^ 22. In coronary artery bypass graft surgery, RIPC was shown to significantly reduce the release of cardiac troponin (cTnT) 6, 12, 24, and 48 h after the surgical procedure^11^^,^ 23. This study aimed to determine the behavior of serum troponin I levels in hemodialysis patients submitted to RIPC associated with irisin expression.

## MATERIALS AND METHODS

A prospective, randomized, double-blind clinical trial was conducted by the Laboratory of Clinical Analysis of ABC Medical School (FMABC), in patients with chronic renal disease under hemodialysis for a period of six months. The procedures followed the principles of the Declaration of Helsinki and were approved by the Research Ethics Committee of Paulista University (UNIP), under protocol no. 2.424.258. Eligibility consisted of patients submitted to outpatient hemodialysis therapy, who were 18 years old or over, and diagnosed with chronic renal failure according to the Kidney Disease Initiative for Global Outcomes (KDIGO). Patients who presented neoplasia, infection, and were HIV+ were excluded. Blood samples were collected to determine troponin I levels using quantitative immunochromatographic methods (Human Quit). IL-6 and TNF-α were analyzed using a chemiluminescent immunoenzymatic method (Simens). Urea and creatinine quantifications were performed using a colorimetric enzymatic method in a fully automated spectrophotometer (COBAS 6000 Roche). Good practices in clinical analysis were followed in all analyses performed in this study. For analysis of gene expression, RNA was extracted using the Trizol^®^ technique. One microgram of cDNA was converted using the Invitrogen Reverse Transcriptase Superscript II RNAse kit, according to the manufacturer’s recommendations.

The qRT-PCR technique was applied to the cDNA sequence obtained so that irisin GADPH, thioredoxin, Nf-kb, GPX4, and selenoprotein could be analyzed as follows: 1X of buffer 2X SYBR Green PCR Master Mix, 2.0 µL of cDNA and 0.4 mM of RT-PCR Primer Assay resulting in 15 µL of final reaction volume. The volume was completed using deionized water. The reaction was run in a Cycler 7500 (Applied Biosystems) using the following program: 95°C for 10 min; 40 cycles of 95°C for 15 s and 60°C for 60 s. The sequence of primers used is presented in Table 1.

**Table 1:** Primers sequence

Gene	Sequence
GSH	FOW 5'CTACGGACCCATGGAGGAG 3'REV 5'AGGCCATGGGACCTTCCT 3'
SE-P	FOW 5'GGTTTGCCTTTTTCCTTCCT 3'REV 5'GCTCCTGGTTGCTGATTCTC 3'
TRX1	FOW 5' GCCATTGGCGATATATTGGA 3'REV 5' CTCTTGACGGAATCGTCCAT 3'
NF-kβ	FOW 5' CTCTGTCATTCGTGCTTCC 3'REV 5' CATCCCATGGTGGACTACC 3'
Irisin	REV 5' GATCCAGCCATCAAGGACAT 3'REV 5'TTGTCCAAGCTAGCATTTCTGA 3'
GAPDH	FOW 5'CTGTGAGGTAGGTGCAAATGC 3'REV 5'GCCCACTTCACCGTACTAACCA 3

The intervention group was submitted to RIPC on the right arm using a sphygmomanometer at 200 mmHg for 5 min, followed by 5 min of deflation, repeated three times for a total of 30 min, during three consecutive hemodialysis sessions. The control group was not submitted to any additional intervention. Blood samples were collected before the start of the first and third weekly sessions. Blood urea nitrogen (BUN) was measured to calculate the single pool Kt/v, and irisin and troponin I to assess cardiac compromise due to hemodialysis. The primary outcome was mortality, while the secondary outcomes were acute myocardial infarction, stroke, and thromboembolic event. Serum levels of TNF-α, selenoprotein, thioredoxin, and NFκB indicated an inflammatory profile. Urine samples were taken to determine the albumin/creatinine ratio and beta-trace protein (BTP) of the first urination. The estimated glomerular filtration rate (eGFR) was calculated using the Modification of Diet in Renal Disease Study equation for adults.

### STATISTICAL ANALYSIS

Qualitative variables are presented as absolute and relative frequency. The Shapiro-Wilk test was used to determine quantitative variables showing non-normal data distribution (*p* < 0.05), and are presented as median values, 25^th^ and 75^th^ percentiles and 95% confidence interval. The Student’s t-test and Wilcoxon test were used to analyze inter-group and intra-group expression of the biomarkers pre- and post-intervention. The Spearman test was used to analyze the correlation between the biomarkers. Stata version 11.0 was used for all analyses.

## RESULTS

This study included 14 hypertensive patients of equal numbers of each sex, 9 (64.3%) of whom were type 2 diabetics (T2DM), aged 44-64 years. There were three deaths due to non-cardiovascular events, one in the intervention group and two in the control group (Table 2). The difference between pre- and post-intervention (RIPC) levels of troponin I were not significant (*p* = 0.28). No significant difference was observed between the pre- and post-collection points for individual biomarkers. In addition, no difference was observed between the RIPC and control groups, except for IL-6 (*p* = 0.039), when analyzing the collection points and the presence or absence of RIPC (Table 3). The Spearman correlation test indicated a significant association (*p* = 0.56) between irisin and troponin I (Table 4).

**Table 2:** Demographic characteristics of the patients

Variables	Patients (n = 14)
Gender (male)	7 (50%)
Hypertension	14 (100%)
Death	3 (21.4%)
Diabetes	9 (64.3%)
Age*	52 (44 - 64)

* Values expressed as mean and 95%CI.

**Table 3:** Association between biomarkers of the group with and without remote ischemic preconditioning (RIPC)

Biomarkers	HD without RIPC	HD with RIPC	*p* *
	Average (95%CI)		
Δ Ureia	64.00 (43.00; 107.80)	61.00 (21.90; 115.80)	0.051
Δ Troponin I	-0.01 (-0.03; 0.05)	0.00 (-0.01; 0.01)	0.361
Δ TNF-α	-0.30 (-3.51; 2.76)	0.55 (-2.69; 9.61)	0.606
Δ IL6	-0.05 (-12.11; 2.54)	5.50 (-1.15; 9.39)	0.039
Δ Irisin	2.29 (-9.90; 14.47)	-1.40 (-14.61; 38.72)	0.698
Δ Tiorredoxin	-1.53 (-23.80; 42.14)	-0.08; (-3.86; 7.53)	0.439
Δ NF-Kβ	-0.66 (-31.81; 42.99)	0.15 (-4.53; 6.95)	0.796
Δ GPX4	1.06 (-2.52; 6.76)	1.26 (-2.55; 12.86)	0.796
Δ Selenoprotein	5.14 (-10.29; 39.05)	0.61 (-12.89; 14.90)	0.439

* Mann-Whitney's; 95%CI: 95% confidence interval.

**Table 4:** Correlation between serum biomarkers

Biomarkers	Irisin		Tiorredoxin		NF-Kβ		GPX4		Selenoprotein	
	Spearman's rho	*p*	Spearman's rho	*p*	Spearman's rho	*p*	Spearman's rho	*p*	Spearman's rho	*p*
Ureia	0.147	0.615	-0.007	0.982	0.323	0.260	0.213	0.464	0.222	0.446
Troponin I	-0.171	0.558	0.082	0.780	0.284	0.325	-0.246	0.396	0.-242	0.405
TNF-α	0.123	0.674	-0.247	0.395	-0.163	0.578	0.223	0.445	0.478	0.084
IL6	0.393	0.164	-0.108	0.714	0.090	0.759	-0.503	0.067	0.125	0.670

## DISCUSSION

This study aimed to establish the relationship between irisin expression and troponin I levels in hemodialysis patients submitted to RIPC. The negative correlation between these variables is in disagreement with the current literature. Hyperglycemic dogs submitted to intravenous dextrose or chemically induced diabetes showed an increase in the extent of myocardial infarction, in addition to annulling the protection resulting from preconditioning^31^. These findings confirm preclinical and clinical evidence that elucidate the adverse interaction between hyperglycemia and cardioprotective pathways. Research conducted on rats, rabbits, dogs, sheep, and humans had very similar findings^24^^-^ 28. This information may explain the lack of correlation found, since our sample was composed of diabetics (64.3%) and hypertensive patients (100%), in contrast to the literature, which consists of 40% diabetics^29^ and 32% hypertensive indivduals^30^. Disparate data likely occurred due to the small sample size.

In diabetic rats, the cardioprotective effect of RIPC was restored, increasing the number of cycles to obtain the desired effect, which indicates that diabetes increases the threshold for preconditioning^31^. In a murine model of ischemia and reperfusion, it was the number and duration of the cycles, rather than the number of limbs exposed to RIPC that determined its effectiveness. The window of early protection disappeared between 1.5 and 2 hours after the end of the stimulus^32^. In a study from 2018, hemodialysis patients submitted to RIPC for three successive sessions did not achieve myocardial protection compared to the control group^33^; however, Park et al. did achieve such protection after twelve sessions^34^. The inconsistency in these findings may be due to the number and/or duration of the sessions.

It has been suggested that poor cardioprotection in diabetics is due to the altered function of the ATP-dependent potassium channel (KATP channel) or the decrease in phosphorylation of important signaling kinases, including Akt (serine/threonine kinase protein) and glycogen synthase (GSK-3)^35^^,^ 36. Currently, prospective data support the possible role of inflammation in diabetogenesis, which is consistent with earlier hypotheses that type 2 diabetes mellitus may be a manifestation of the acute cytokine-mediated response of the innate immune system^37^. In this scenario, positive associations were observed between IL-6 and PCR that remained after adjusting for body mass index, family history of diabetes, smoking, exercise, alcohol use, and hormone replacement therapy. The multivariate relative risks for the highest versus the lowest quartiles were 2.3 for IL-6 (95%CI, 0.9-5.6, p tending toward = 0.07) and 4.2 for PCR (95%CI, 1.5-12.0, p tending toward = 0.001)^38^^,^ 39.

Regarding mortality in the proposed six-month follow-up period, three (21.4%) non-cardiovascular deaths occurred, one in the intervention group and two in the control group. This corroborates data regarding the duration of hemodialysis that ascertained the relationship between the duration of hemodialysis therapy and mortality as a primary outcome in 11 countries; mortality rate (deaths/100 patient-years) was 16.9% (95%CI, 16.2-17.6) for a period of 121-365 days^40^. In all countries, mortality was higher at baseline compared with the intermediate period, but the intermediate and late periods were similar. Within each period, a higher mortality occurred in the United States compared to most other countries. Thus, internationally, the initial period of hemodialysis constitutes the period of high risk in the countries studied, and substantial differences in mortality have been reported among these. In conclusion, independent of the time of collection, RIPC did not modify the levels of irisin and troponin I, even though both are known biomarkers.
